# Functional Genomics Approach for Identification of Molecular Processes Underlying Neurodegenerative Disorders in Prion Diseases

**DOI:** 10.2174/138920212801619223

**Published:** 2012-08

**Authors:** Urmila Basu, Le Luo Guan, Stephen S Moore

**Affiliations:** 1 Department of Agricultural, Food and Nutritional Science, University of Alberta, Edmonton, Alberta, Canada T6G 2P5; 2 The University of Queensland, Centre for Animal Science, Queensland Alliance for Agriculture and Food Innovation, St. Lucia, 4072, Queensland, Australia

**Keywords:** Prion, TSEs, gene expression, functional candiate genes, PRNP, microarray.

## Abstract

Prion diseases or transmissible spongiform encephalopathies (TSEs) are infectious neurodegenerative disorders leading to death. These include Cresutzfeldt-Jakob disease (CJD), familial, sporadic and variant CJD and kuru in humans; and animal TSEs include scrapie in sheep, bovine spongiform encephalopathy (BSE) in cattle, chronic wasting disease (CWD) of mule deer and elk, and transmissible mink encephalopathy. All these TSEs share common pathological features such as accumulation of mis-folded prion proteins in the central nervous system leading to cellular dysfunction and cell death. It is important to characterize the molecular pathways and events leading to prion induced neurodegeneration. Here we discuss the impact of the functional genomics approaches including microarrays, subtractive hybridization and microRNA profiling in elucidating transcriptional cascades at different stages of disease. Many of these transcriptional changes have been observed in multiple neurodegenerative diseases which may aid in identification of biomarkers for disease. A comprehensive characterization of expression profiles implicated in neurodegenerative disorders will undoubtedly advance our understanding on neuropathology and dysfunction during prion disease and other neurodegenerative disorders. We also present an outlook on the future work which may focus on analysis of structural genetic variation, genome and transcriptome sequencing using next generation sequencing with an integrated approach on animal and human TSE related studies.

## 1 INTRODUCTION

Prion diseases, or transmissible spongiform encephalopathies (TSEs), are invariably fatal neurodegenerative disorders affecting both humans and animals. Human prion diseases include Kuru, Creutzfeldt-Jakob disease (CJD) for about 85% of the cases, familial CJD in 10-15% of the cases and variant CJD which is limited to the UK and France [1, 2]. Animal TSEs (Table **1**) include scrapie in sheep, bovine spongiform encephalopathy (BSE) in cattle, chronic wastings disease (CWD) in cervids, transmissible mink encephalopathy in mink, and feline spongiform encephalopathy (FSE) in cats, and exotic ungulate spongiform encephalopathy (EUE) of captive wild ruminants [3]. Animals affected by TSEs increase the risk of transmission to humans as well as seriously decline the farm animal production [4]. For example, since the BSE epidemic in 1986, slaughtering of millions of cattle and import ban on the beef have resulted in substantial losses. Prion diseases occur as sporadic, genetic, and transmissible disease, however, sporadic and heritable forms of the diseases are more frequent. 

Ingestion of contaminated biological material via food resulted in the acquired forms of diseases e.g., Kuru was the first known human TSE due to ritualistic cannibalism [5]. BSE is another massive common-source epidemic caused by meat and bone meal fed primarily to dairy cows [6, 7] and more recently vCJD in human has been associated with exposure to the BSE agents [1]. On the other hand, mutations in the normal prion protein encoded by the PRioN Protein (PRNP) gene, are linked to genetically inherited prion diseases including Gerstmann-Strausler-Sheinker (GSS) disease, fatal familial insomnia (FFI) and genetically associated CJD [2, 4].

## 2 PATHOGENESIS OF PRION DISEASE

In mammals, the conversion of the normal, cellular isoform of the prion protein (PrP^C^) to the disease-causing isoform (PrP^Sc^) is the key process underlying prion diseases. PrP^C^ and PrP^Sc^ have distinct conformations with PrP^C^ has extensive α-helical content whereas PrP^Sc^ is rich in β-sheet structure with less α-helical content [8, 9], has the propensity to aggregate and has the resistance to proteolysis [10]. Prion diseases in animals can be characterized by PrP^Sc^ replication and accumulation, spongiform vacuolation and astrocytic gliosis, synaptic degeneration leading to neurodegeneration and lethality [11] while these symptoms are common in many neurodegenerative disorders [5, 12]. Approximately 45% of the PrPC protein is α-helical with two very short stretches of β-sheets and its conversion to PrPSc results in protein with ~30% α-helix and 45% β-sheet. Other ancillary proteins are also involved in this conversion process and PrPc appears to bind to PrPSc to form an intermediate complex [13]. The major route of transmission for many TSEs is through oral infection after which prions spread through the gut to secondary immune tissues and then to the brain [5]. Furthermore, prions have been detected in different lymphoid organs including the spleen, lymph nodes, Peyer’s patches and tonsils [14-16].

Prion protein (PrP) is encoded by a chromosomal gene, *PRNP, *with a single exon for the PrP open reading frame although the gene itself comprises of two to three exons [17-20]. The other exons of the PRNP gene contain untranslated sequences including the promoter and termination sites with multiple copies of GC-rich repeats in the PrP promoter [21]. Comparison of a total of 937 *PRNP* sequences from 83 species suggested a striking degree of conservation among the mammalian sequences [22]. However, variations in PrP sequences exist both between species and between individuals within species greatly affecting susceptibility to prion infection [23]. Increasing evidence suggests that other genes in addition to the PRNP genes also contribute to the genetic susceptibility of acquired TSEs, thus there is a need to improve our understanding of the molecular mechanisms underlying prion disease pathogenesis. Genome-wide studies in cattle [24-28], sheep [29, 30], mice [31, 32], and humans [33] have identified genomic regions and positional candidate genes, other than the prion gene, involved in TSE pathogenesis. This review highlights the recent advances in the field of prion diseases in human, mouse models and ruminant species to understand the complexities of molecular pathways using high throughput functional genomics technologies.

## 3 HIGH THROUGPUT GENE EXPRESSION STUDIES ASSOCIATED WITH HUMAN PRION DISEASES

The *PRNP *gene determines both susceptibility and phenotypes of prion diseases in humans, with point mutations leading to a specific pathological phenotype [34, 35]. A genome-wide study in patients with various forms of prion diseases (variant, sporadic, iatrogenic CJD and kuru patients) confirmed that the risk of developing prion diseases is strongly associated with the polymorphic codon 129 of the *PRNP *gene [33]. Presence of single nucleotide polymorphisms (SNPs) in the intron of *PRNP*, retinoic acid receptor-β protein* (RARB)*, and SCG10/stathmin-like 2, a neuronal growth-associated protein (*STMN2*) increased the risk of prion diseases. Retinoic acid is known to regulate the expression of the prion protein in cell culture [36], and SCG10 modulates microtubule stability in neuronal cells, which, in turn, might potentially modulate prion neurotoxicity [37] indicating the involvement of these genes in the pathogenesis of prion disease.

Different approaches including human genome wide association studies (GWAS), mouse mapping and differential expression studies have suggested the association of Shadoo (Sho, shadow of prion protein)**(SPRN) and E3 ubiquitin ligase (HECTD2) with risk of sporadic and variant CJD [38-40]. Shadoo is a neuronally expressed glycoprotein of unknown function. Due to a conserved physiological activity between PrP^C^ and Sho, it might be acting on similar signaling pathways in human prion pathobiology [41]. By homology, HECTD2 is an E3 ubiquitin ligase and is involved in the ubiquitinylation of proteins for targeted degradation by the proteasome [40]. The ubiquitin-proteosome system has also been implicated in prion diseases and a number of other neurodegenerative disorders [40].

In another study [42], microarray analysis of the frontal cortex of 15 patients with sporadic CJD revealed the involvement of 79 up-regulated (e.g., metallothionein-1 and 2) and 275 down-regulated genes (e.g., Synaptosomal-associated protein 25 (SNAP25) modulating synaptic function and plasticity) compared to healthy controls. Metallothionein-1 and -2 are cysteine-rich intracellular proteins with a high capacity to bind to zinc and copper ions and are known to be mainly present in the cytoplasm of astrocytes of the cerebral cortex and white matter [43]. Altered expression of genes involved in metal ion binding activity would suggest an aberrant ion homeostasis in CJD [42]. SNAP-25 is involved in modulating synaptic function and plasticity and the synaptic impairment is a major pathological mechanism of CJD [44]. Further characterization of these genes is crucial to understanding the molecular basis of pathological process of prion diseases.

## 4 IDENTIFICATION OF CANDIDATE GENES THROUGH FUNCTIONAL GENOMIC STUDIES IN ANIMAL PROTEIN DISEASES

It has been well documented that besides PRNP, other genes are also involved in the pathogenesis of prion diseases [45]. Large scale gene expression profiling of infected vs. control animals to identify differentially expressed (DE) genes may help in understanding novel genes and pathways that are switched on and off at various time points during the pathogenesis of prion diseases. Different functional genomics technologies have been used to detected differential gene expression including cDNA libraries [46], mRNA differential display [47], suppression subtractive hybridization [48], microarrays [49-54] and more recently next generation sequencing [55]. These studies in experimental animal models or from clinically-infected animals have revealed multiple genes and signaling pathways that may be involved in TSE pathogenesis.

### 4.1 Prion Related Gene Expression Changes in Animal Models

Due to the availability of limited material from prion-infected samples at pre-clinical and clinical stages for humans and other animals; and due to the similarities between the mouse and human genome, the mouse models have been used in several prion pathogenesis studies. These models have provided the numbers and replication required for designing large scale studies to identify the genes of interest at different time points throughout disease pathogenesis [56]. Furthermore, mouse models can also be used for testing the candidate genes from over-expressing transgenics to knockouts, to determine their effect on prion pathology.

In brain tissues from experimental scrapie-infection animal models (Table **2** provides a list of high throughput studies conducted in mouse models), differentially expressed (DE) genes have been identified at different stages of prion diseases using microarray analysis [47, 57-60]. The global analysis of the overall transcriptional response in pre-clinical and clinical central nervous system tissue of mice in response to prion infection revealed that many of the individual genes identified had an association with the neurodegenerative process in prion disease as well as Alzheimer's disease (AD) [57]. The differential expression of genes involved in cellular stress (oxidative stress and ER stress), activated ER and mitochondrial apoptotic pathways, protease inhibitors, calcium binding proteins, endosome/lysosome function, immunity, synapse function, metal ion binding, and activated cholesterol biosynthesis, have been shown in the scrapie-infected hippocampus [58] and brain [59, 60]. Furthermore, all these studies have indicated similarities between gene expression patterns found in brains affected by AD and aging, respectively.

The differential expression of the genes involved in immunity, protein folding, ubiquitin/proteosome or in the endosome/lysosome system was observed in the brain and the spleen in scrapie-infected mice prior to the onset of clinical symptoms [61]. Altered expression of four genes, ATPase, Na+/K+ transporting, beta 1 polypeptide (Atp1b1), Growth hormone (Gh), acidic leucine-rich nuclear phosphoprotein 32 family member A (Anp32a) and Granulin (Grn) at the very early time of post-infection provided insights into the mechanism of pathogenesis [61]. Hwang *et al.* [62] performed a detailed characterization using two prion strains, different incubation times, and mice from six different genetic backgrounds and identified a core of 333 genes central to prion disease that were differentially expressed in all five of the combinations involving mice with normal levels of prion protein. Transcriptional analysis of follicular dendritic cells and macrophage enriched splenic cells revealed the genes related to iron metabolism and homeostasis as the major pathways [63]. Genome wide expression study in mice inoculated with BSE homogenate indicated changes in two main biological processes, neural cell metabolism and defense mechanisms [64]. Some of the genes identified in these studies may serve as markers for prion disease diagnosis which could be putative candidates for drug therapies. 

### 4.2 Prion Related Gene Expression in Ruminants

Gene expression profiling studies in natural target ruminant species (cattle, sheep, elk/deer) infected by natural route (oral infection) are important for understanding the pathogenesis of the prion diseases. In cattle, orally infected with BSE agent (12 and 45 months post-infection), 101 DE genes in Peyer’s patch tissues [52] and 176 DE genes in medulla [53, 54] have been identified. These genes are mainly associated with the synapse function (e.g., tachykinin, synuclein, neuropeptide Y, cocaine, amphetamine-responsive transcript, and synaptosomal-associated protein 25 kDa); calcium ion regulation (e.g., parvalbumin, visinin-like, and cadherin); immune and inflammatory response (major histocompatibility complex (MHC) class II), and apoptosis (cholinergic receptor).

Another study [65] investigated the effect of prion pathogenesis on gene expression in cattle using microarray and 114 genes related to immune response, apoptosis, cell adhesion, stress response, and transcription were found to be differentially regulated (Table **3**). Due to inherent limitations of microarrays including sequence-specific probe hybridization, background and cross-hybridization of related genes, digital gene expression (DGE) tag profiling using next generation sequencing was used to compare the transcriptomic profiles of medulla tissues from cattle infected with BSE [55]. This study identified 190 DE transcripts from different pathways including neuroactive ligand–receptor interaction, regulation of the actin cytoskeleton, focal adhesion, SNARE interactions in vesicular transport, T-cell receptor signaling, calcium signaling, TGF-beta signaling, and MAPK signaling. Inaddition, the Tag profiling was successful in identifying additional pathways like ErbB signaling, the T cell receptor, the Wnt signaling, antigen processing, cytokine-cytokine receptor interaction, Gap junction, and the PPAR signaling as compared to the previous microarray studies on BSE-infected medulla tissues [53, 55]. The common DE genes detected in all these studies [52-55, 65] on cattle were: S100 calcium binding and Calmodulin; Prolactin-related protein; GTPase, IMAP family member, Histocompatibility complex, class II, Metallopeptidase and Myosin, Glutathione S transferase A, Aldo-Keto reductase family and Nuclear receptor subfamily group H.

Up-regulation of three chaperones including endoplasmic reticulum (ER) chaperones, Grp94 and Grp170 has strongly suggested the presence of ER stress and the activation of the unfolded protein response (UPR) in BSE-infected cattle [66]. The patterns of gene expression in white blood cells following oral infection of cattle with Bovine amyloidotic spongiform encephalopathy (BASE) has also been investigated and significant change in expression of genes linked to T- and B-cell development and activation, and to inflammatory responses was observed [67]. High-density whole genome association study in 143 BSE case and 173 control animals elucidated chromosomal regions and positional candidate genes associated with disease [28]. These positional candidate genes include hypothetical gene LOC521010, similar to FK506 binding protein 2 encoding a protein from immunophilin protein family with a role in protein folding, leucine-rich repeats and immunoglobulin-like domains 2, LRIG2, a protein known to be involved in protein-protein interactions, and Repulsive guidance molecule family member A, a glycosyl phosphatidylinositol -anchored glycoprotein, with a role as an axon guidance protein in the central nervous system [28]. 

Recently, transcriptomics analysis in the caudal medulla oblongata of scrapie-symptomatic sheep using a CVI custom designed 4x44K microarray platform identified 148 genes (Table **3**) that are primarily associated with the immune response, ion transport, cell adhesion, and transcription [68]. Transcriptome analysis of scrapie-infected lymph nodes and spleen tissues from sheep have revealed the repression of genes linked to inflammation and oxidative stress, and the up-regulation of genes related to apoptosis [69]. We performed first comprehensive microarray analysis of high-throughput gene expression associated with CWD disease using bovine specific oligos. The differential expression of a set of key genes from different pathways from multiple organs of CWD infected elk include major regulatory and signaling networks, neuronal signaling, synapse function in neurological disease, calcium signaling, apoptosis and cell death, and immune cell trafficking and inflammatory response [70].

In this review, we have summarized the emerging role of functional genomic technologies in identifying genetic changes which could be used as potentially diagnostic biomarkers for risk determination and as general indicators of disease progression. Most of the expression profiling studies performed so far are in different models after clinical symptoms appear, however, prion diseases and other neurodegenerative disorders have a long pre-symptomic phase [71]. Additional studies will be necessary to investigate differential gene expression through full transcriptomic analysis from animals at both pre-clinical and clinical time points to identify new genes, proteins and pathways involved in the pathogenesis of TSEs in order to select gene(s) which could be used as a pre-clinical diagnostic marker. 

## 5 PRION DISEASES AND OTHER NEURODEGENERATIVE DISORDERS

Mis-folding of the prion protein and its progressive accumulation into aggregates is the common feature of many major neurodegenerative diseases, like AD, Parkinson's disease (PD), fronto-temporal dementia, Huntington's disease (HD) which is shared by TSEs or prion disorders like CJD [72, 73]. In all these neurological diseases, there is continued synthesis of mutant, aggregation-prone proteins such as tau (in Taupathies), α-synuclein (in PD), polyglutamine-containing proteins (in HD), and amyloid-β (in AD) for many decades before neuropathology symptoms are evident [13]. Furthermore, these proteins or protein aggregates indicate ‘prion-like’ phenomenon of CJD and BSE and have been shown to spread to other cells and brain regions resulting in the progressive deterioration [74, 75]. Prion diseases and AD share some common morphological and pathophysiological features including the copper binding properties of the amyloidogenic proteins, oxidative stress and the evidence of free radical damage, the formation of amyloid plaques and neurofibrillary pathology [13, 76]. Histological and biochemical studies [77, 78] have recently suggested that Alzheimer’s disease and TSE pathologies synergistically interact with the possibility of a direct interaction between the proteins leading to cross-seeding and increased pathogenesis with the possibility of molecular cross talk between both diseases. This interaction may disrupt interaction between PrP^c^ and a co-receptor impairing the neuron’s signal-transduction pathways leading to amyloid β oligomerization outside the cell and intracellular accumulation of tau protein leading to synaptic plasticity, axonal damage and neuronal death [77-79]. 

In addition to the common histopathological features, oxidative stress Fig. (**1**) is an important factor that has been implicated in the pathogenesis of a number of neurodegenerative disorders [58]. The misfolding of proteins or the development of plaques is followed by loss of synapses and possible abnormalities in mitochondrial function. Increased sensitivity to oxidative stress in neurodegenerative diseases is through alteration of anti-oxidant enzyme functions, and increased lipid peroxidation resulting in impairment of anti-oxidant response [80]. There is increasing evidence that secondary mitochondrial dysfunction by inhibition of several key enzymes in the mitochondrial energy production pathway or through the triggering of caspase-dependent apoptosis occurs in a number of major neurodegenerative diseases [81]. In addition, oxidative stress and apoptosis in prion diseases results in endoplasmic reticulum stress through the disruption of calcium homeostasis in the cell causing calcium overload in the cytoplasm leading to synaptic loss and neuronal death [82]. Other studies have also revealed that the overall level of oxidative damage to proteins, lipids and DNA may result in ubiquitin-proteasome dysfunction leading to neuronal degeneration [83]. Since the molecular pathways involved in different neurodegenerative diseases are similar, further studies to explore these molecular interactions, using high throughput functional genomics technologies combined with new bioinformatics capabilities, will provide a new conceptual framework for the pathogenesis of different neurodegenerative diseases and will have important therapeutic implications. 

## 6 FUTURE DIRECTIONS

Until now, most of the functional genomics studies have employed microarrays and genome wide association studies to identify potential candidate genes or pathways involved in prion disease in different models or organisms. With the recent advances in next generation sequencing, exome sequencing, large scale data integration and data mining, it should be possible to investigate other key regulators including miRNA, and transcription factors playing a role in the pathogenesis mechanisms [84]. Some preliminary studies are beginning to establish the role of miRNA in prion diseases. The first comprehensive microarray analysis of miRNA expression in the brains of mice infected with mouse-adapted scrapie reported the de-regulation of 15 miRNAs controlling a number of genes and signaling pathways during prion induced neurodegeneration [85]. Another study provided evidence of up-regulation of miRNA-146a in sporadic Creutzfeldt-Jakob disease (sCJD) and Gerstmann-Straussler-Scheinker syndrome (GSS) suggesting its role in innate immune response and antiviral immunity [86]. Forthcoming directions of research will focus on a co-ordinated approach to link transcriptome and microRNAome data with extensive computational analysis, next generation sequencing studies spanning the entire genome or exome, analysis of structural genetic variation (copy number variations through deletions, duplications, and inversions) and genome wide association analysis. All these studies Fig. (**2**) will provide new insights of the regulatory networks involved in prion pathogenesis of human and animal prion diseases. Prions are also emerging as ‘extreme case of epigenetic inheritance’ and prion based mechanisms may influence genetic information [87]. Understanding of the epigenetic information in addition to the genetic information is becoming important for the preventive diagnosis and treatment of diseases [88]. The availability of next generation sequencing technologies will empower the study of epigenetic variations including DNA methylation, histone modifications, and chromatin accessibility which may be involved in prion associated neurodegenerative disorders. Furthermore, studies using functional proteomics and new physiology techniques such as laser capture microdissection on subpopulations of cells in pre- and post-symptomics phases of prions and other neurodegenerative diseases will complement the genomics studies to determine selective biomarkers.

## Figures and Tables

**Fig. (1):**
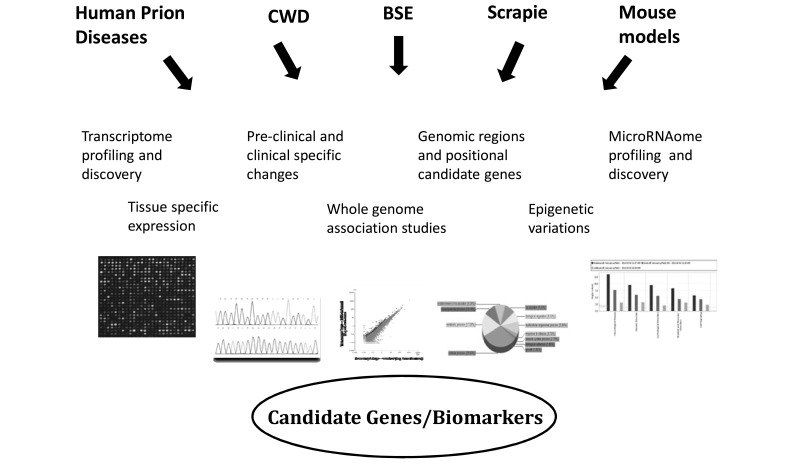
Involvement of oxidative stress in prion diseases and other neurodegenerative diseases leading to mitochondrial dysfuntion, endoplasmic reticulum stress through altered gene functions.

**Fig. (2):**
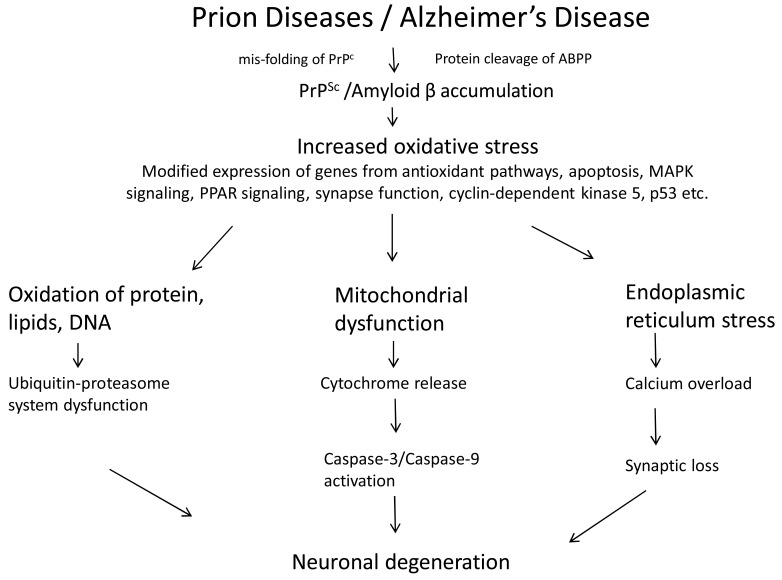
Understanding of complex prion diseases: Identification of genes and genetic markers through functional genomics.

**Table 1.** Transmissible Spongiform Encephalopathies (Prion Diseases) in Humans and Animals

	Type of Disease	Pathogenesis Mechanism / Mode of Transmission
***Human Diseases***		
Kuru	Infectious	Through Cannibalism
Sporadic CJD	Unknown	Spontaneous Conversion to PrP^C^ to PrP^Sc^ Due to Spontaneous Mutation
Familial CJD	Genetic	PRNP Mutations
Latrogenic CJD	Infectious	Infection from Prion-Contaminated Material
Variant CJD	Infectious	Consumption of Infected Animals
***Animal Diseases***		
Scrapie (Sheep and Goats)	Infectious	Exposure to Infected Sheep
Transmissible Mink Encephalopathy	Infectious	Infection from Prion Contaminated Feed
Bovine Spongiform Encephalopathy (BSE)	Infectious	Infected Meat and Bone Meal
Chronic Wasting Disease (CWD)	Infectious	Contaminated Pasture
Exotic Ungulate Spongiform Encephalopathy (EUE)	Infectious	Infection from Prion Contaminated Material
Feline Spongiform Encephalopathy (FSE)	Infectious	Infection from Prion Contaminated Feed

**Table 2.** High Throughput Gene Expression Studies in Different Tissues from Prion-infected Mouse Model Leading to the Identification of Major Functional Candidate Genes / Markers / Pathways

Study	Tissue	Technology Used	Functional Candidate Genes / Markers / Pathways
Dandoy-Dron *et al.*, 1998 [47]	Brain from scrapie-infected mouse	mRNA differential display	Cathepsin S, the C1q B-chain of complement, apolipoprotein D, scrapie-responsive gene (*ScRG-1*) and *ScRG-2*
Booth *et al.*, 2004 [57]	Brain from scrapie-infected mouse	cDNA microarray	158 differentially expressed (DE) genes Functional groups - secreted extracellular proteins, lysosomal proteases, defense and immune response-related proteins, signal transduction genes and cell growth, biogenesis-related genes
Brown *et al.*, 2005 [58]	Hippocampal tissue from scrapie-infected mouse	Affymetrix high-density oligonucleotide probe arrays	78 DE genes- sterol-C4-methyl oxidase and sterol-C5-desaturase (cholesterol biosynthesis), complement component C1qβ, short coiled-coil protein, signal recognition particle 9, THUMP domain-containing 1, neurofilament-L
Riemer *et al.*, 2004 [49]	Cortex, medulla from scrapie-infected mouse	Mouse Genome U74Av2 arrays	114 DE genes- proteinase inhibitor 2 (SPI-2); α-2-macroglobulin, lipocalin 24, CCAAT/ enhancer-binding protein delta (C/EBPdelta)
Xiang *et al.*, 2004 [59]	Brain from scrapie-infected mouse	Affymetrix Mouse Expression Arrays	121 DE genes - several members of the cathepsin family, protease inhibitors, S100 calcium binding proteins
Skinner *et al.*, 2006 [60]	Brain from scrapie-infected mouse	cDNA microarrays	400 DE genes - chemokine (C-X3-C) receptor 1, CD9 antigen, ATPase Na+/K+ transporting beta 1 polypeptide, cathepsin B, glial fibrillary acidic protein, and apolipoprotein E
Sorensen *et al.*, 2008 [51]	Brain from multiple scrapie infected mouse	cDNA microarrays	349 prion-related genes (PRGs)- transforming growth factor (*TGF*)-beta 1; extracellular signal-regulated kinase-mitogen-activated protein kinase (ERK/MAPK) signaling; transcription regulators, Early Growth responsive protein (EGR1) and CAMP responsive element binding protein 1 (CREB1)
Kim *et al.*, 2008 [61]	Brain and spleen from multiple scrapie infected mouse	Affymetrix microarray	67 DE genes - prolactin (*Prl*), Gh, and pro-opiomelanocortin-alpha (*Pomc1*); ATPase Na+/K+ transporting beta 1 polypeptide (*Atp1b1*); Acidic leucine-rich nuclear phosphoprotein 32 family member A (*ANP32A)*
Hwang *et al.*, 2009 [62]	Eight distinct mouse strain-prion strain combinations	Microarray Subtractive analysis	A core of 333 genes central to prion disease
Huzarewich *et al.*, 2011 [63]	Spleen from scrapie-infected mouse enriched for dendritic cells and macrophages	Mouse genome 4x44K version microarray	1753 DE genes- Leucine-rich proteoglycan decorin (*DCN*); osteoglycin (*OGN*), proline arginine-rich and leucine-rich repeat protein (*PRELP*), and chondroadherin (*CHAD*)
Tortosa *et al.*, 2011 [64]	Transgenic mice overexpressing bovine cellular prion protein (PrPc)	Mouse Genome 430 2.0 arrays	87 DE genes- Neuronal PAS domain protein 3 (Npas3, a transcription factor involved in the neuronal signaling]) and ribonucleotide reductase M2 B (Rrm2b, related to DNA)

**Table 3.** High Throughput Gene Expression Studies in Prion-infected Different Tissues from the Ruminants Leading to the Identification of Major Functional Candidate Genes / Markers / Pathways

Study	Animal/Tissue	Technology Used	Functional Candidate Genes / Markers / Pathways
Khaniya *et al.*, 2009 [52]	BSE-infected cattle Peyer’s patch	Microarray	90 DE genes- Major histocompatibility complex (*MHC*) class II, MHC class II DQ alpha, leukocyte-derived arginine aminopeptidase (*L-RAP*)
Tang *et al.*, 2009 [65]	BSE-infected cattle brains	Microarray	114 DE genes- immune response, apoptosis, cell adhesion, stress response, and transcription- S100 cal­cium binding and Calmodulin; Prolactin-related protein; GTPase, IMAP family member, Histocompatibility complex, class II, Metallopeptidase; Myosin; Glutathione S transferase A; Aldo-Keto reductase family; Nuclear receptor subfamily group H
Tang *et al.*, 2010 [66]	BSE-infected cattle brains	Microarray	230 DE genes- immune response, apoptosis, cell adhesion, ER stress related response and transcription; ubiquitin-proteasome pathway; autophagy-lysosome system-glucose-regulated protein 94 (Grp94/gp96); glucose-regulated protein 170 (Grp170/Orp150); Inositol 1,4,5-triphosphate receptor (IP3, ER calcium-depletion stress); reticulon 1, 3 and 4 (ER stress induced apoptosis)
Almeida *et al.*, 2011a, b [53, 54]	BSE-infected cattle caudal medulla tissues	Microarray	176 DE genes- extracellular matrix (ECM) receptor, cell adhesion, neuroactive ligand–receptor interaction, SNARE interactions in vesicular transport, and MAPK signalling pathway synapse pathway - tachykinin, synuclein, neuropeptide Y, cocaine, amphetamine-responsive transcript, and synaptosomal-associated protein 25 kDa (*SNAP25*); calcium ion regulation (parvalbumin, visinin-like, and cadherin)
Basu *et al.*, 2011 [55]	BSE-infected cattle caudal medulla tissues	Tag profiling Solexa sequencing	190 DE genes- neuroactive ligand-receptor interaction, regulation of the actin cytoskeleton, focal adhesion, SNARE interactions in vesicular transport, T-cell receptor signaling pathway, Calcium signaling pathway, TGF-beta signaling pathway, MAPK signaling pathway *SNAP25*, vesicle-associated membrane protein 1, MHC class II, DQ alpha 5, thrombospondin 1, guanine nucleotide exchange factor isoforms
Panelli *et al.*, 2011 [67]	Bovine amyloidotic spongiform encephalopathy infected cattle white blood cells	Microarray	56 DE genes- T- and B-cell development and activation, inflammatory responses
Filali *et al.*, 2011 [68]	Scrapie-infected sheep caudal medulla tissues	4x44K microarray	350 DE genes- immune response, ion transport, cell adhesion, and transcription- calpain 6, galanin 1 and pancreatitis associated protein 1; three downregulated (collagen 1 α2, collagen 3 α2) and melatonin receptor 1b (*MTNR1B)*
Gossner *et al.*, 2011 [69]	Scrapie-infected sheep lymph nodes and spleen	Microarray	52 DE genes in lymph nodes and 37 DE genes in spleen Up-regulation of genes related to apoptosis and repression of genes linked to inflammation and oxidative stress
Basu *et al.*, 2012 [70]	Brain, midbrain, thalamus, spleen, RPLN and tonsil of CWD-infected elk	Microarray using Bovine-specific oligos	329 DE genes in the brain, 249 DE genes in the spleen, 30 DE genes in the retropharyngeal lymph node (RPLN) and 55 DE genes in the tonsil - neuronal signaling and synapse function, calcium signaling, apoptosis and cell death and immune cell trafficking and inflammatory response
